# Survey of Predatory Coccinellids (*Coleoptera: Coccinellidae*) in the Chitral District, Pakistan

**DOI:** 10.1673/031.007.0701

**Published:** 2007-01-26

**Authors:** Inamullah Khan, Sadrud Din, Said Khan Khalil, Muhammad Ather Rafi

**Affiliations:** ^1^ National Agricultural Research Council, Islamabad, Pakistan; Department of Plant Protection, NWFP Agricultural University, Peshawar, Pakistan

## Abstract

An extensive survey of predatory Coccinellid beetles (Coleoptera: Coccinellidae) was conducted in the Chitral District, Pakistan, over a period of 7 months (April through October, 2001). A total of 2600 specimens of Coccinellids were collected from 12 different localities having altitudes from 1219.40–2651.63 m. Twelve different species belonging to 9 genera of 3 tribes and 2 sub-families were recorded. Two sub-families, viz, Coccinellinae Latreille, 1807 and Chilocorinae Mulsant, 1846 were identified. The following 8 species belonged to family Coccinellinae Latreille 1807 and tribe Coccinellini Latreille 1807: *Coccinella septempunctata* Linnaeus, 1758, *Hippodamia* (*Adonia*) *variegata* Goeze, 1777, *Calvia punctata* (Mulsant, 1846), *Adalia bipunctata* (Linnaeus, 1758),*Adalia tetraspilota* (Hope, 1831), *Aiolocaria hexaspilota* Hope 1851, *Macroilleis* (*Halyzia*) *hauseri* Mader, 1930,*Oenopia conglobata* Linnaeus, 1758. Only one species namely *Halyzia tschitscherini* Semenov, 1965 represented tribe Psylloborini of the sub-family Coccinellinae Latreille, 1807. Three species occurred from sub-family Chilocorinae Mulsant 1846 and tribe Chilocorini Mulsant 1846: *Chilocorus rubidus* Hope, 1831, *Chilocorus circumdatus* (Gyllenhal, 1808), *Priscibrumus uropygialis* (Mulsant, 1853). From the aforementioned species 6 were recorded for the first time from Pakistan: *Chilocorus circumdatus, Calvia punctata, Adalia bipunctata, Macroilleis* (*Halyzia*) *hauseri, Priscibrumus uropygialis*, and *Oenopia conglobata*.

## Introduction

Coccinellids or ladybirds, members of the family Coccinellidae, are among the most familiar beetles and have common names around the world, such as lady cows, God's cows and virgin's insect (Moreton 1969). These are small to medium size beetles with an oval, oblong or hemispherical body shape (Majerus 1994). Most of them are of bright shining colors with a pattern of spots or patches against a contrasting background. Many appear to be distasteful to birds, and their conspicuous appearance is an example of warning coloration (Moreton 1969).

Numerous species of Coccinellids are major biological agents of pests such as aphids, mealybugs, scale insects, thrips and mites in all parts of the world (Moreton 1969, Hawkeswood 1987 and Majerus 1994, Khan 2001). Some are specific in their food choice, while many are polyphagous. The introduction of the vedalia ladybird, *Rodolia cardinalis* Mulsant, from Australia into California in 1888 to control cottony cushion scale, *Icerya purchasi*, which threatened the citrus industry, is widely regarded the most successful instances of biological pest control (Majerus 1994).

**Figure 1:**
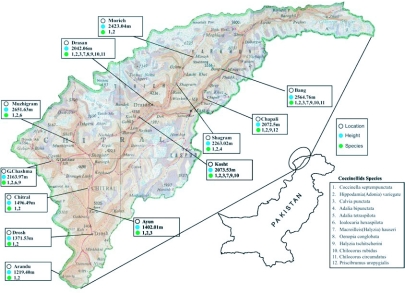
Locations of coccinellid species collected in the Chitral District, Pakistan

The family Coccinellidae comprises 5,200 described species worldwide (Hawkeswood 1987). Fleming (2000) reported 4,000 predatory species of Coccinellid including more than 300 species from Indo-Pak Subcontinent, Irshad (2001) listed 71 species of Coccinellids in Pakistan. The present study was undertaken to explore and prepare an inventory of the predacious Coccinellid, found at different altitudes in Chitral district at 7112 and 7353 east longitude and between 3513 and 3655 north latitude at the extreme northern territory of Pakistan (Figure 1). This covers an area of 14,850 km2, while the total cultivated area was 22,552 hectares (Anonymous, 1999). Ninety nine percent population of Chitral is engaged in farming. Eighty percent of the farmers possess less than two hectares and only one percent has 2.5 hectares of land. Wheat, maize, pulses, fodder, fruit and vegetables are the major crops (Anonymous, 1999). The climate of Chitral is temperate. Temperatures range from 15 to 45°C (Anonymous, 1999). The whole district Chitral has many rare species of flora and fauna, most of which are endangered.

## Materials and Methods

The study was conducted to collect predatory coccinellids from 12 localities at different altitudes (1219.40 to 2651.63 m) in the Chitral District. Each locality was repeatedly sampled throughout 6 month (April through October, 2001). Samples were collected from a wide variety of terrestrial habitat throughout in each locality to ensure that the overall landscape of that locality was represented. Several collecting methods were used, depending on the type of habitats sampled. Adult specimens were collected by sweep-net, aspirator and hand picking. In some localities more then one method was used for insect collection. Adult insects collected from various habitats were killed in a cyanide bottle and pinned. Each specimen was tagged with the information about host plants, locality, and date. To protect the specimens from the insect pests, naphthalene tablets were added to collection boxes. Immature stages were collected directly from the habitats and preserved in 70% ethyl alcohol in bottles. Each bottle was labeled with information of host, area and date from which it was collected. The Chitral District is divided six regions (Tehsil). Two sites in each region were chosen. These are shown in Table 1 along with the altitude of each site.

**Table 1.**
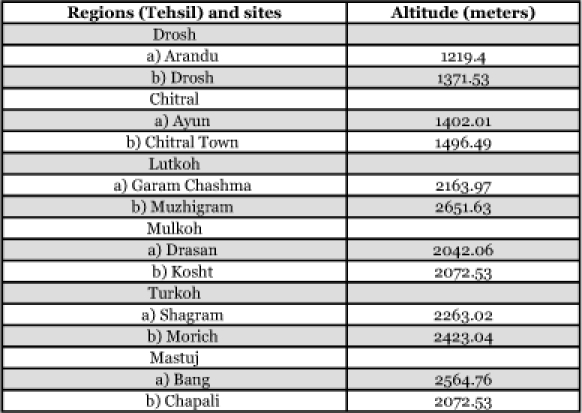
Locations and altitudes of collection sites.

## Identification of Specimens

Field collected beetles and immature stages of these adults were taken to the National Agricultural Research Center (NARC) Islamabad, Pakistan where they were identified to species level using published literature. To confirm identification some of the collected species were sent to the Universita degli studi di Pavia, Centro Interdisciplinare di Bioacustica e Ricerche Ambientali, Via Taramellia 24-27100 Pavia, Italy.

**Table 2.**
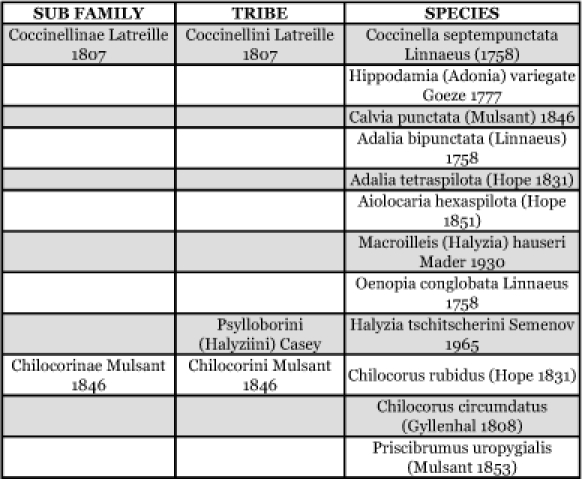
Coccinellid species collected from the Chitral District, Pakistan.

## Results and Discussion

Twelve species of predatory beetles belonging to three different tribes (Chilocorini, Coccinellini and Psylloborini), and two sub-families, Coccinellinae Latreille 1807 and Chilocorinae Mulsant 1846 of the Coccinellidae family, were identified from the Chitral District, Pakistan (Figure 1, Table 2). All identified species were recorded for the first time from Chitral District, and of these six were recorded for the first time from Pakistan.

### Family:*Coccinellinae* Latreille Tribe:*Coccinellini* Latreille *Coccinella septempunctata* Linnaeus

*Coccinella septempunctata* was found in all localities from altitudes ranging from 1219.40 to 2651.63 m. It was collected while feeding on aphids and scale insects on trifolium, maize and wheat. *C. septempunctata* was previously reported from Faisalabad (Laylpur) by a number of authors (Rehman 1940; Khan and Sultan 1949; Gillani 1976). Mohyudddin (1981) and Shah (1983) recorded *C. septempunctata* from Peshawar on a variety of plants including *Hibiscus esculantus, Solanum melongena, Lactuca sativa* and *Glycin max*. Irshad (2001) found this predacious species on various insect pests including *Aleurocanthus husaini* Corbett, *Aleurocanthus woglumi* Ashby, *Aleurolobus barodensis* Mask., *Acyrhsiphon pissum* Harris, *Aphis craccivora* Koch, *Aphis fabae* Theobald, *Aphis gossypii* Glov., *Bemesia tabaci* Genn, *Centrococcus insolitus* Green, *Chaitophorus* spp., *Diaphorina citri* Kuw., (Psyllidae: Homoptera), *Dialerodes citri* Ashm., *Dialerodes elongata* (Daz,) (Dialeorididae: Homoptera), *Ferrisina virigata* (Ckll), *Lipaphis pseudobrassicae* (Davis), *Macrosiphum rosaephormis* Das., *Myzus persicae* (Sluz), *Neomaskellia* spp. (Aleyrodidae: Homoptera), *Pyrilla purposilla* (Fulgorridae: Homoptera), *Quadraspidiotus pernicious* Comst. (Diaspididae: Homoptera), *Rhopalosiphum maidis* (Fitch) (Aphididae: Homoptera), and *Spilococcus spp*. (Pseudococcidae: Homoptera). Therefore, it was conformed that *C. septempunctata* Linnaeus (1758), is a generalized predator and widely distributed throughout Pakistan.

### *Hippodamia (Adonia) variegate* Goeze

The second most frequent species after *C. septempunctata* was *Hippodamia* (*Adonia*) *variegate*, which was found in all the selected localities and at all altitudes (1219.40–2651.63 m). This species was also collected from trifolium, wheat and maize crops. Gillani (1976) previously reported it from Faisalabad, Mohyuddin (1981) from different locations in Pakistan, and Shah (1983) recorded it on *Trifolium alexenderium, Cucumis melo* and *H. esculantus*, where it was feeding on aphid species, Irshad (2001) examined this species feeding on various insect and mite pests, i.e., *Adelges joshhi* (Adelgidae: Homoptera), *Anuraphis helichrysi* Katt, *Acrythosiphon pisum* Harris, *Aphis craccivora* Koch, *Therioaphis trifolii* Monell, (Aphididae: Homoptera) *Schizaphis graminum* Rond, *Macrosiphum graminum* (Hby), *R. maidis* Fitch, *Hasura* sp. (Asterolecaiidae: Homoptera), *Drosihca magneferae* (Green) (Margoridae: Homoptera), *Dioryctria abietella* (Schiff.), (Pyrallididae: Lepidoptera), *Tetranychus atlanticus* Mcg. *Tetranychus* sp. (Tetranychidae: Acari) with a wide distribution of Pakistan. Our results are in conformity with data collected by other authors.

### *Calvia punctata* (Mulsant)

*Calvia punctata* was recorded for the first time during this study and is an addition to Coccinellid fauna of Pakistan. It was collected from Drosh, Drasan, Kosht and Bang sites from an altitude of 1524.00 m to above 2133.60 m. During collection, this species was found feeding on scale insects on walnut tree and other wild vegetation. *C. punctata* was found in four morphological types. Two different species of the genus *Calvia* (*Calvia sykesi* Crotch and *Calvia bretti* Marder) were reported by Canepari et al. (1997) from India, Nepal and Himalayas.

### *Adalia bipunctata* (Linnaeus)

This species was collected from the Shagram site, at 2263.02 m in the Turkoh region on *Triticum aestivum* and *T. alexanderium* feeding on wheat aphids. This species is also a new addition to Coccinellid fauna of Pakistan.

### *Adalia tetraspilota* (Hope)

*Adalia tetraspilota* was found from Chitral Town(1496.49 m) and Drasan (2042.06 m) sites. It was collected from different vegetation preying on scale insect pests. This species was also reported from Murree feeding on *Adelges* spp., *Q. perniciosus* and *D. abietella* by Irshad (2001) and from Nepal by Canepari et al. (1997).

### *Aiolocaria hexaspilota* (Hope)

*Aiolocaria hexaspilota* was collected from Garam Chashma and Kandujal in the Lutkoh region at altitudes ranging from 2163.97 to 2651.63 m. This species was collected while feeding on scale insects on walnut and other vegetation, Irshad (2001) recorded this species on *Q. perniciosus*, from Northern Pakistan, and from Nepal by Canepari et al. (1997).

### *Macroilleis* (*Halyzia*) *hauseri* Mader

During this survey *Macroilleis* (*Halyzia*) *hauseri* was discovered from the Drasan and Kosht sites of the Mulkoh region from 2042.06 to 2438.40 m above sea level. This species was also a new entry to the reported Coccinellinid fauna of Pakistan. It was found preying on *Q. perniciosus* Comst.

### *Oenopia conglobata* Linnaeus

*Oenopia conglobata* was reported for the first

time from Pakistan. It was found attacking aphid species on wheat in the Drasan site. Kuznetsov (1997) earlier reported this species from far eastern Russia.

### Tribe: Psylloborini (Halyziini) Casey

#### *Halyzia tschitscherini* Semenov

*Halyzia tschitschenni* was collected from walnut trees while feeding on scale insects at altitudes of more than 1828.80 m from the Drasan, Kosht and Bang sites. Mohyudddin (1981) previously recorded this species from Chitral District, Pakistan. This species was also reported by Alia (2002) from Poonch district of Azad and Jamu Kashmir, while other species of this genus, *Halyzia sanscrita* (Mls) and *Halyzia straminea* were also reported by Canepari et al. (1997) from Nepal.

**Family:*Chilocorinae* Mulsant**

**Tribe:*Chilocorini* Mulsant**

### *Chilocorus rubidus* (Hope)

*Chilocorus rubidus* was retrieved from *Q. perniciosus* from the Drasan site at height of about 2042.06 m. It was collected feeding on scale insects on apricot trees, Irshad (2001) examined this species on *Eulecanium tiliae* (L) *Q. perniciosus* Comst, *Licanium* p. and *Parlatoria* sp. from Abbotabad, Peshawar and Parachinar. While Canepari et al. (1997) reported *Chilocorus rubidus* Hope, from Nepal during survey of Himalayas.

### *Chilocorus circumdatus* (Gyllenhal)

Only one specimen of *Chilocorus circumdatus* was recorded from the Drasan site. It was found feeding on scale insects on apricot. *C. circumdatus* is a new entry in the reported Coccinellid fauna of Pakistan. Smith (1995 and 1998) reported this species from Queensland, Australia along with citrus pests, *Coccus viridis, Polyphagotarsonemus latus* and *Aonidiella aurantii* in citrus orchard. Canepari et al. (1997) also recorded this species in Nepal.

### *Priscibrumus uropygialis* (Mulsant, 1853)

*Priscibrumus uropygialis* (Mulsant) was collected while feeding on *Q. perniciosus* on apple and pear orchards in the Chapali site at altitude of about 2072.53 m. This species was recorded for the first time during this study and therefore the first reported from Pakistan. However Kovar (1995) and Canepari et al. (1997) reported *Priscibrumus uropygialis* from India and Nepal.

## Conclusion

The present study was the first attempt to describe the coccinellid fauna of the Chitral District. The object of this study was to explore, identify and prepare inventory of predatory coccinellid species in the Chitral District, which will be helpful for the future researchers working on predatory coccinellid species of this region. For this purpose a preliminary survey was carried out in the six regions of the Chitral District. According to the results 12 different species from 9 genera belonging to sub-families Coccinellinae and Chilocorinae existed in the area. The species were, *Coccinella septempunctata*, *Hippodamia* (*Adonia*) *variegata, Calvia punctata, Adalia bipunctata, Adalia tetraspilota, Aiolocaria hexaspilota, Macroilleis* (*Halyzia*) *hauseri, Oenopia conglobata, Halyzia tschitscherini, Chilocorus rubidus, Chilocorus circumdatus* and *Priscibrumus uropygialis*.

Further survey is needed of those areas that were not covered in this study to fully explore predatory coccinellids fauna of district Chitral. The total area of the Chitral District is approximately 1480 km^2^, which is mostly mountainous. These mountains are bare except for lower areas and cultivation is practiced only in small patches at the bottom of the deep and narrow valleys. The altitude of the mountains range from 1066.75 m in the extreme south in Arandu to 7690 m at mount Terichmir. A survey in such an area is not easy, where only camels or other animals used for transportation. However, efforts were made to survey evenly in distract Chitral to cover the maximum area.

## Note

Paper copies of this article will be deposited in the following libraries. Senckenberg Library, Frankfurt Germany; National Museum of Natural History, Paris, France; Field Museum of Natural History, Chicago, Illinois USA; the University of Wisconsin, Madison, USA; the University of Arizona, Tucson, Arizona USA; Smithsonian Institution Libraries, Washington D.C. U.S.A.; The Linnean Society, London, England.
